# Altered Protein Expression in the Ileum of Mice Associated with the Development of Chronic Infections with *Echinostoma caproni* (Trematoda)

**DOI:** 10.1371/journal.pntd.0004082

**Published:** 2015-09-21

**Authors:** Alba Cortés, Javier Sotillo, Carla Muñoz-Antoli, Bernard Fried, J. Guillermo Esteban, Rafael Toledo

**Affiliations:** 1 Departamento de Parasitología, Facultad de Farmacia, Universidad de Valencia, Burjassot, Valencia, Spain; 2 Centre for Biodiscovery and Molecular Development of Therapeutics, Australian Institute of Tropical Health and Medicine, James Cook University, Cairns, Queensland, Australia; 3 Department of Biology, Lafayette College, Easton, Pennsylvania, United States of America; McGill University, CANADA

## Abstract

**Background:**

*Echinostoma caproni* (Trematoda: Echinostomatidae) is an intestinal trematode that has been extensively used as experimental model to investigate the factors determining the expulsion of intestinal helminths or, in contrast, the development of chronic infections. Herein, we analyze the changes in protein expression induced by *E*. *caproni* infection in ICR mice, a host of high compatibility in which the parasites develop chronic infections.

**Methodology/Principal Findings:**

To determine the changes in protein expression, a two-dimensional DIGE approach using protein extracts from the intestine of naïve and infected mice was employed; and spots showing significant differential expression were analyzed by mass spectrometry. A total of 37 spots were identified differentially expressed in infected mice (10 were found to be over-expressed and 27 down-regulated). These proteins were related to the restoration of the intestinal epithelium and the control of homeostatic dysregulation, concomitantly with mitochondrial and cytoskeletal proteins among others.

**Conclusion/Significance:**

Our results suggests that changes in these processes in the ileal epithelium of ICR mice may facilitate the establishment of the parasite and the development of chronic infections. These results may serve to explain the factors determining the development of chronicity in intestinal helminth infection.

## Author Summary

Intestinal helminth infections are among the most prevalent parasitic diseases and about 1 billion people are currently infected with intestinal helminths. Incidence of intestinal helminth infections is high due to both socio-economic factors that facilitates continuous re-infections and the lack of effective vaccines. In this context, further knowledge on the host-parasite relationships is required to elucidate the factors that determine the expulsion of the intestinal helminths or, in contrast, the chronic establishment of the infections. *Echinostoma caproni* (Trematoda) is an intestinal trematode that has been extensively used as experimental model to investigate these factors. Depending on the host species. *E*. *caproni* is rapidly rejected or develops chronic infections. Herein, we analyze the changes in protein expression induced by *E*. *caproni* infection in a host in which the parasites develop chronic infections. These data may serve to get a better understanding of the factors determining the development of chronic intestinal infections. A total of 37 spots were identified differentially expressed. These proteins were related to the restoration of the intestinal epithelium and the control of homeostatic dysregulation, mitochondrial and cytoskeletal proteins among others. This suggests that the changes in these processes in the intestinal mucosa may facilitate the development of chronic infections.

## Introduction

Intestinal helminth infections are among the most prevalent parasitic diseases. Recent studies have estimated that about 1 billion people are currently infected with at least one species of intestinal helminth mainly in developing regions of Asia, Africa and Latin-America [1]. Although intestinal helminths rarely kill their human hosts, they commonly cause chronic or recurrent infections that have an important impact in health. The most common symptoms are related to effects on nutrition causing growth retardation, malabsorption syndrome and vitamin deficiencies or impaired cognitive function [2,3]. Additional abnormalities such as intestinal obstruction, chronic dysentery, rectal prolapse, respiratory complications, iron-deficiency anemia or debilitating disease can also appear [4–6]. Moreover, parasitic helminth infections in livestock are responsible for significant economic losses due to decreases in animal productivity and the cost of anthelminthic treatments of parasitized individuals [1].

About 40 million people are infected with food-borne trematodes, including members of the family Echinostomatidae, mainly in East and Southeast Asia [7]. Echinostomes are cosmopolitan parasites that infect a large number of different warm-blooded hosts, both in nature and in the laboratory. About 20 species belonging to nine genera of Echinostomatidae are known to cause human infections around the world [8,9]. They constitute an important group of food-borne trematodes of public health importance with prevalences that ranges from 3% in some areas of Asia [10,11]. Apart from their interest in human health echinostomes, and particularly *Echinostoma caproni*, have been used for decades as experimental models to the study of food-borne trematodes—vertebrate host relationships [12,13]. *E*. *caproni* is an intestinal trematode with no tissue phases in the definitive host [13]. After infection, the metacercariae excyst in the duodenum and the juvenile worms migrate to the ileum, where they attach to the mucosa [13]. *E*. *caproni* has a wide range of definitive hosts, although its compatibility differs considerably between rodent species on the basis of worm survival and development [12]. In mice and other hosts of high compatibility, the infection becomes chronic, while in hosts of low compatibility, (such as rats) the worms are expelled from the 2–4 weeks post-infection (wpi) [14,15]. Moreover, the consequences of the infection in each host class are markedly different. The establishment of chronic infections in CD1 mice is dependent upon a local Th1 response with elevated production of IFN-γ [16]. The infection induces important inflammatory responses, a marked epithelial injury and a rapid increase of iNOS expression [15–17]. Concomitantly with these events, chronic infections impair the processes of renewal of the intestinal epithelium inducing elevated levels of crypt-cell proliferation and tissue hyperplasia at the site of the infection [18]. In contrast, the early rejection of *E*. *caproni* is associated with the development of a local Th2/Th17 phenotype and changes in the tissue structure are not observed [16,19]. Because of these characteristics, the *E*. *caproni*-rodent model is extensively used to elucidate several aspects of the host-parasite relationships in intestinal infections, such as the induction of distinct effector mechanisms and their effectiveness in parasite clearance.

Comparative proteomic studies allow to obtain a broad view of the changes induced by a particular process, as in this case, the establishment of intestinal infections. Herein, we analyze the alterations in the protein expression induced by the *E*. *caproni* infections in the ileum of a host of high compatibility in which chronic infections are developed. This information may be useful to gain a better understanding on the factors that facilitate the development of chronic infections with intestinal helminthes and the consequences of helminth infections in hosts chronically infected.

## Material and Methods

### Animals and infection procedures

The present study was performed using male CD1 mice weighing 30–35 g. The strain of *E*. *caproni* employed and the infection procedures have been described previously [20]. Briefly, encysted metacercariae of *E*. *caproni* were removed from kidneys and periacardial cavities of experimentally infected *Biomphalaria glabrata* snails and used for infection. A total of 16 mice were each infected by gastric gavage with 75 metacercariae of *E*. *caproni*. Additionally, 16 mice were left uninfected and used as uninfected s. All the animals were sacrificed at 2 weeks post-infection (wpi) to obtain tissue samples.

### Ethics statement

The animals were maintained under conventional conditions with food and water *ad libitum*. This study has been approved by the Ethical Committee of Animal Welfare and Experimentation of the University of Valencia (Ref#A18348501775). Protocols adhered to Spanish (Real Decreto 53/2013) and European (2010/63/UE) regulations.

### Cell collection and protein extraction

Ileal sections from uninfected and infected mice were removed at necropsy and intestinal epithelial cells (IEC) were isolated as described before [21]. In brief, the ileal sections were opened longitudinally and rinsed by gentle shaking in washing buffer: ice-cold Hank’s balance salt solution (HBSS) containing 2% of heat-inactivated fetal calf serum (FCS). Supernatant was then removed and fresh washing buffer was added to the ileal sections. This step was repeated at least 4 times, until the supernatant was clear. The tissue was then cut into small 1 cm-long segments and incubated for 20 min at 37°C in HBSS containing 10% FCS, 1nM EDTA, 1mM DTT, 100 U/ml penicillin and 100 μg/ml streptomycin (dissociation buffer). The supernatant was collected and maintained on ice and the incubation was repeated a second time with fresh dissociation buffer. Supernatants were combined and filtered through a 100 nm cell strainer before IEC were pelleted out by a centrifugation at 200 g for 10 min at 4°C and washed three times in PBS under the same centrifuge conditions to remove any residual medium.

Protein extraction was performed using the M-PER Mammalian Protein Extraction Reagent (Thermo Scientific) according to the manufacturer’s instructions. Shortly, M-PER Mammalian Protein Extraction Reagent was added to the IEC pellet (20:1, v/v), mixed by vortex and incubated at room temperature (RT) for 20 min with continuous gentle agitation. The lysate was then clarified by centrifugation at 18,000 g for 15 min at 4°C, transferred into a new tube and stored at -80°C until use.

### Preparation of biological replicates and protein labeling

In order to increase the biological significance and avoid erroneous conclusions due to individual variations, four biological replicates were performed for each experimental group (uninfected and infected). Each biological replicate was obtained by pooling the same amount (20 μg) of protein extracted from the IEC isolated from four different mice. Then, 50 μg of protein from each biological replicate were cleaned and precipitated with the 2D Clean-up Kit (GE Healthcare), pellets were resuspended in 18 μl of a proper buffer (25 mM Tris, 7 M urea, 2 M thiourea, 4% CHAPS, pH 8,5), and proteins were fluorescently tagged with CyDye DIGE Fluor minimal dyes (GE Healthcare), following manufacturer’s instructions. One microliter of dye (400 pmol) was added to each sample and maintained on ice for 30 min in the dark. The reaction was stopped by adding 1 μl of 10 mM lysine. To minimize any dye-specific labeling artefacts, two biological replicates of each experimental group (infected and uninfected) were labeled with Cy3 and the other two were labeled with Cy5. The internal standard, prepared by mixing the same amount of protein of each sample included in the experiment, was always labeled with Cy2.

### Two-dimensional differential in gel electrophoresis (2D-DIGE)

Ileal protein extracts from *E*. *caproni*-infected and uninfected mice were compared across four 2D-DIGE gels to identify proteins significantly modulated by the presence of the parasite. The four pairs of Cy3- and Cy5-labeled biological replicates (50 μg of protein each) were combined with a 50 μg aliquot of the Cy2-labeled internal standard. The mixtures containing 150 μg of protein were then separated in the first dimension, i.e. isoelectric focusing as second dimension were run following previously described protocols using the isoelectric focusing protocol for 24 cm Immobiline Drystrips. The IPG strips (24 cm, nonlinear pH 3–11) where rehydrated overnight with rehydration buffer (8 M urea, 4% CHAPS, 1% ampholytes and 12 μl/ml of DeStreak™), and the labeled samples were then applied to the strips by anodic cup loading, after the addition of DTT and ampholytes up to a final concentration of 65 mM and 1%, respectively. The isoelectric focusing was carried out at 20°C in the Ettan IPGphor 3 System (GE Healthcare) as follows: (I) 300 V for 4 h; (II) gradient to 1,000 V for 6 h; (III) gradient to 8,000 V for 3 h; and (IV) 8,000 V up to 32,000 Vh. Prior to the second dimension the strips were equilibrated in two steps, 15 min each, in equilibration buffer (50 mM Tris, 6 M urea, 30% glycerol and 2% SDS) containing either 2% DTT or 2.5% iodoacetamide, respectively. The separation of proteins in the second dimension was performed on an Ettan DALTsix system (GE Healthcare) using 12.5% polyacrylamide gels. Electrophoresis was run at 1 W/gel for 1h followed by 5 h, approximately, at 15 W/gel.

### Imaging and 2D-DIGE data analysis

Gels were scanned in a Typhoon 9400 Variable Mode Imager (GE Healthcare) at appropriate wavelengths for each fluorophore: Cy2 (488/520 nm), Cy3 (532/580 nm) and Cy5 (633/670 nm), and at 50 μm resolution. The non-essential information was removed using ImageQuant Tools software and DeCyder v7.0 software was employed for image analysis. The differential in gel analysis module was used for automatic spot detection and abundance measurements in each individual gel, comparing the normalized volume ratio of each spot from a Cy3- or Cy5-labeled sample to the corresponding Cy2 signal from the internal standard. The data sets were collectively analyzed using the biological variation analysis module of the same software, which allows inter-gel matching and calculation of standardized average volume ratios (AVRs) for each protein spot among the 4 gels of the study. Statistical analysis was assessed for each change in AVR using Student’s *t* test and false discovery rate (FDR). Statistical significance was considered when *p*<0.05 and *q*<0.05, respectively. Moreover, inter gels matching of statistically different spots was confirmed manually.

Unsupervised principal components analysis (PCA) and hierarchical clustering analysis (HCA) (Euclidean) were performed using the DeCyder extended data analysis module, both on all protein spots that were present in the 4 gels of the experiment (100% presence) and the group of spots identified as significantly modified as a consequence of the infection. These multivariate analyses clustered the individual biological replicates based on a collective comparison of expression patterns from the set of proteins chosen, with any *a priori* knowledge of the biological reasons for clustering.

### Mass spectrometry (MS) and protein identification

The protein spots showing greater changes in their expression levels were manually excised from the gel and washed twice with double-distilled water. Thereafter, proteins were reduced in 100mM ammonium bicarbonate containing 10 mM DTT for 30 min at 56°C, alkylated with iodoacetamide 55 mM in 100 mM ammonium bicarbonate for 20 min at RT in the dark and, finally, digested in-gel with an excess of sequencing grade trypsin (Promega) overnight at 37°C, as described before [22]. Protein digestion was stopped with 1% trifluoroacetic acid (TFA) and peptides were dried in a vacuum centrifuge and resuspended in 7 μl of 0.1% TFA, pH 2. One microliter of peptide mixture was spotted onto a MALDI target plate and allowed to air dry at RT before adding 1 μl of matrix, a 5 mg/ml solution of α-cyano-4-hydroxy-transcinnamic acid (Sigma) in 0.1% TFA and 70% acetonitrile (ACN), and left to air dry again.

The samples were analyzed in a 5800 MALDI TOFTOF (AB Sciex) in positive reflectron mode using 3000 laser shots per position. Previously, the plate and the acquisition methods had been calibrated with 0.5 μl of CM5 calibration mixture (AB Sciex), in 13 positions. For the MS/MS analysis, 5 of the most intense precursors were selected for each position, according to the following threshold criteria: a minimum signal‐to‐noise of 10; a minimum cluster area of 500; a maximum precursor gap of 200 ppm and a maximum fraction gap of 4. MS/MS data was acquired using the default 1kV MS/MS method. Several spots could not be identified by MALDI TOFTOF, however, so liquid chromatography and tandem mass spectrometry (LC-MS/MS) was performed. Five microliters of each sample were loaded onto a trap column: NanoLC Column, 3 μ C18-CL, 350 μm x 0.5 mm (Eksigen) and desalted with 0.1% TFA at 3 μl/min for 5 min. The peptides were then loaded onto an analytical column: LC Column, 3 μ C18-CL, 75 μm x 12 cm (Nikkyo), equilibrated with 5% ACN, 0.1% formic acid (FA). Elution was carried out with gradient of 5 to 45% B in A for 15 min (A: 0.1% FA; B: ACN, 0.1% FA) at a constant flow rate of 300 nl/min. Peptides were analyzed in a mass spectrometer nanoESI qQTOF (5600 TripleTOF, AB Sciex). The tripleTOF was operated in information-dependent acquisition mode, in which a 0.25-s TOF MS scan from 350–1250 m/z was performed, followed by 0.05-s product ion scans from 100–1500 m/z on the 50 most intense 2–5 charged ions.

### Database search

Both MS-MS/MS and LC-MS/MS data were sent to MASCOT 2.5 (Matrix Science) via ProteinPilot (AB Sciex) and database search was performed on NCBInr (non-redundant) database with taxonomy set to Metazoa. Searches were done with tryptic specificity, allowing one missed cleavage and a tolerance on mass measurement of 100 ppm in MS mode and 0.8 Da for MS/MS ions. Carbamidomethylation of cysteine was used as fixed modification and oxidation of methionine and deamination of asparagine and glutamine as variable modifications. A protein identification was considered accurate when at least three peptides were identified with an overall MASCOT score greater than 50.

Functional classification and intracellular localization of the identified proteins were assessed using the KEGG Pathway (http://www.genome.jp/kegg/pathway.html) and UniProtKB resource (http://www.uniprot.org/). A cytoscape plugin, the Biological Networks Gene Ontology (GO) tool (BiNGO 2.3) was used to identify overrepresented biological processes GO terms [23]. Settings for BiNGO included using a hypergeometric test with a significance threshold of 0.05. The *P*-values were corrected for multiple testing by the Benjamini & Hochberg correction.

## Results

### *E*. *caproni* induces rapid changes on protein expression profile in the ileal epithelium of infected mice

A 2D-DIGE proteomic analysis was performed on whole ileal cell extracts from eight biological replicates corresponding to *E*. *caproni*-infected and uninfected mice (4 replicates each) and 2D-gel images were then subjected to computational analysis using the DeCyder software (S1 Fig). Both univariate and multivariate statistical analysis indicated that *E*. *caproni* infection induces an intense remodeling of protein expression pattern in the ileal mucosa of mice early after the establishment of the infection.

### 2D-DIGE analysis of infection-induced changes

**Fig 1:**
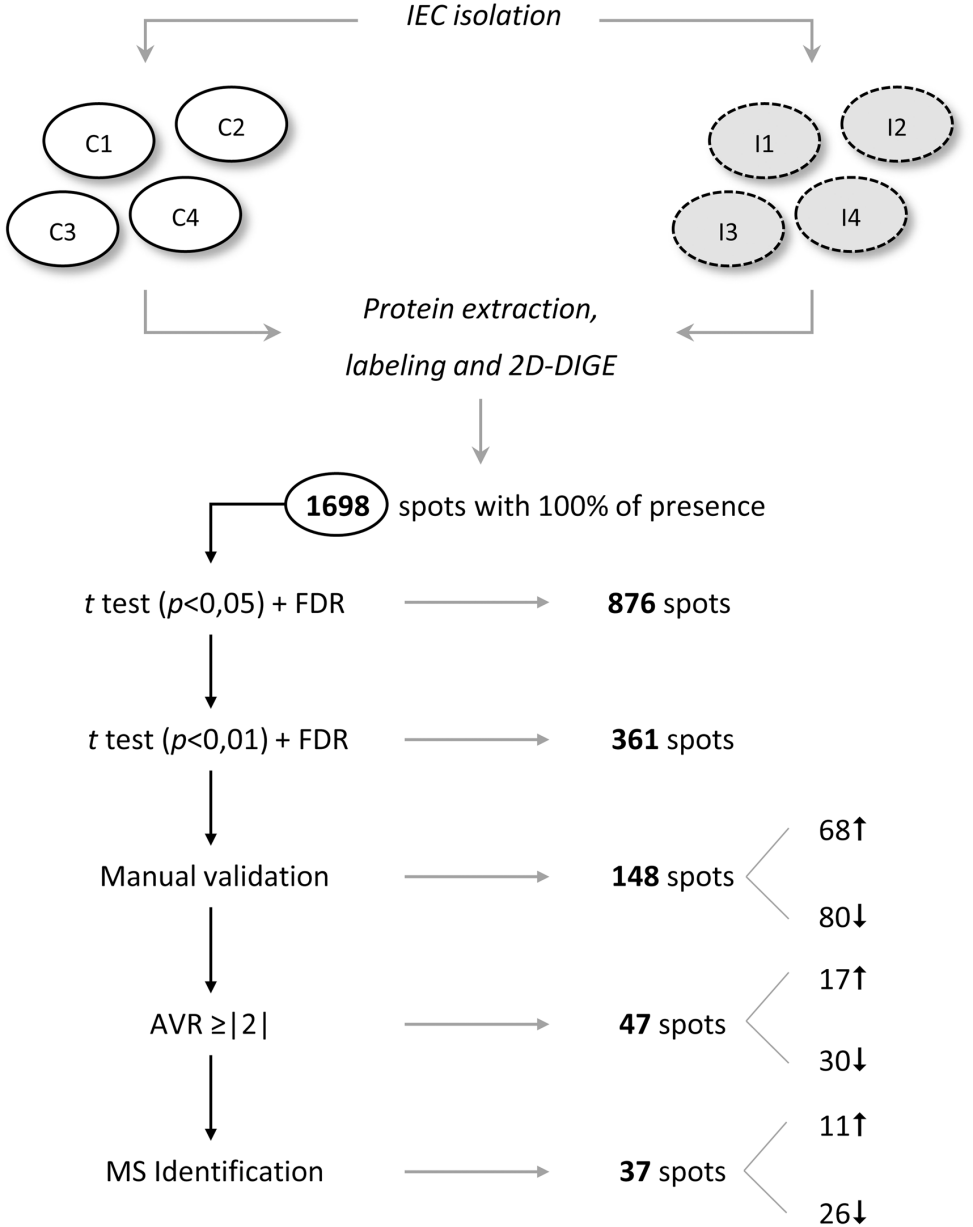
Schematic overview of the results obtained in the 2D-DIGE assay and the selection criteria applied. Those spots displaying greater and most significant differential expression between the ileum of *Echinostoma caproni*-infected and uninfected mice were selected. FDR, false discovery rate; AVR, average volume ratio.

The inter-gel spot matching, carried out using biological variation analysis module, revealed a total of 1,698 well defined spots with a 100% of presence, i.e. found in each gel included in the 2D-DIGE experiment (Fig 1). The average abundance of each spot was then calculated and significant changes were evaluated. A total of 876 spots, representing a 51.6% of the total number of spots, showed significant variations within a 95% confidence interval in both Student’s t test (*p*<0.05) and FDR (*q*<0.05). In view of the large number of differentially expressed proteins, different selection criteria were sequentially applied in order to select for protein identification those spots whose expression was mostly affected as a consequence of the infection. In a first step, the confidence interval in the Student’s test was reduced to 99%, yielding a total of 361 spots showing a value of *p*<0.01. To guarantee the proper comparison of spots among gels, the correspondence of these 361 spots among all the gels were manually validated, and 148 were unambiguously confirmed (68 up-regulated in the ileum of infected mice and 80 down-regulated). Forty-seven of these spots showed an AVR greater than or equal to |2.00| in the four gels analyzed (17 overexpressed and 30 downregulated at 2 wpi). Finally, 37 of these spots (11 and 26 up and downregulated, respectively) could be extracted from the gel and successfully identified by MS. Fig 1 summarizes the results of applying the consecutive selection filters from the initial set of spots with 100% of presence to those that were eventually identified by MS.

### Multivariate statistical analysis

**Fig 2:**
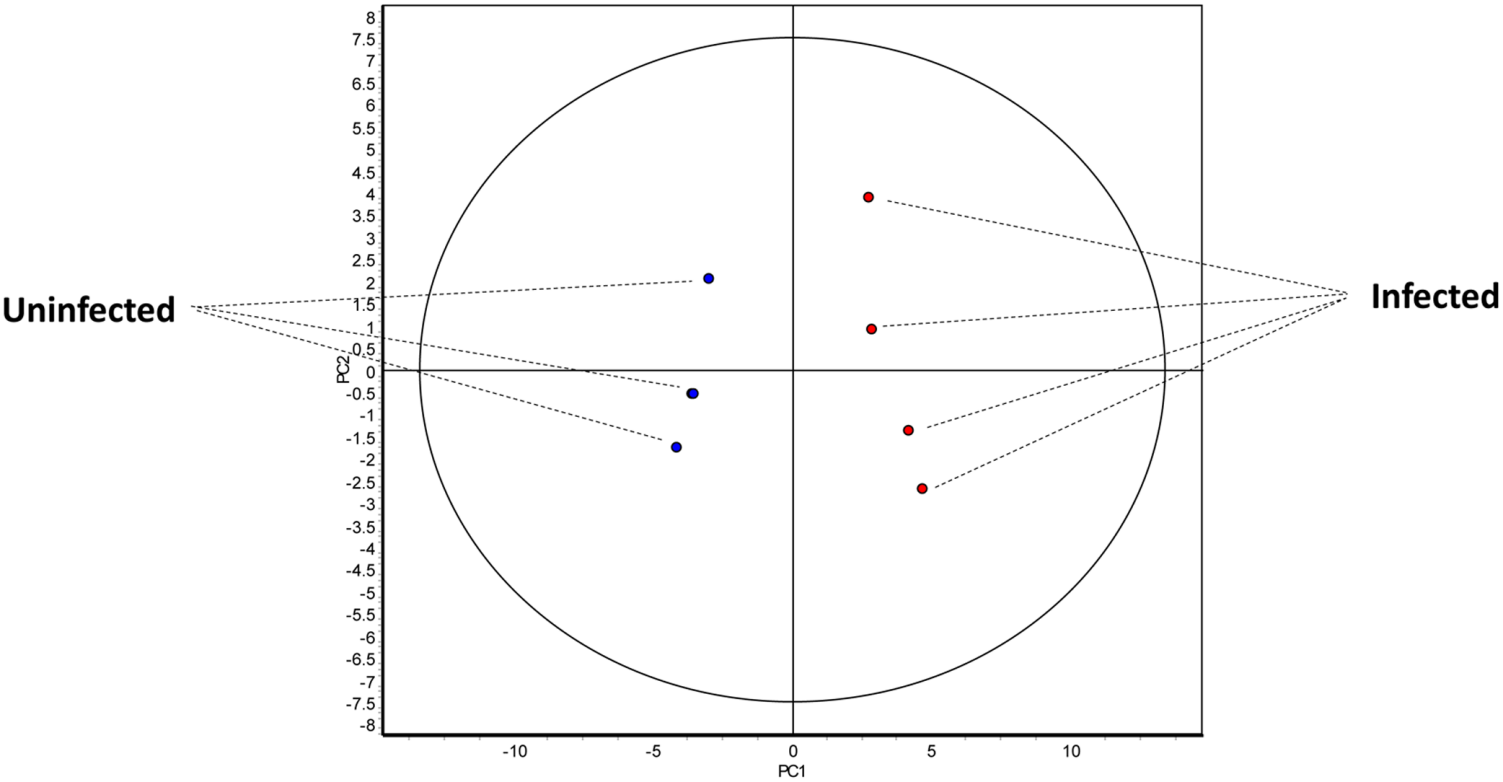
Multivariate statistical analysis applied to the set of 1,698 spots with 100% of presence in the 2D-DIGE experiment. An indication about clustering and trends in protein expression profiles in the ileum of *Echinostoma caproni*-infected and uninfected mice is given. 2-dimensional score plot from the principal component analysis of the 8 individual biological replicates (*E*.*caproni*-infected in red and uninfected in blue).

In order to establish the biological significance of the infection-induced protein changes, multivariate statistical tests were performed on the proteins identified by 2D-DIGE. PCA and HCA were carried out on both the total number of spots with 100% presence in the experiment (1,698) and those displaying larger significant statistical differences between uninfected and infected mice (361 spots). As shown in Fig 2, both PCA and HCA applied to the set of 1,698 spots with 100% presence were able to separate graphically the two groups of samples. In the PCA the two groups were separated in the basis of the first principal component. Moreover, all biological replicates were within the range of normality (95% of confidence), discarding the existence of outliers among the samples (Fig 2). Similarly, HCA applied to the same set of protein spots grouped the replicates in two main categories according to their condition of infected or uninfected. The heat diagram shows that protein expression patterns displayed by uninfected and infected animals were clearly different, suggesting that *E*. *caproni* infection induces a significant change on IEC (S2 Fig). In this first analysis, however, the proteins could not be clustered according to their expression pattern indicating the existence of a wide variability when the spots are compared individually (S1 Fig). This is not strange since both, significantly and non-significantly differentially expressed spots were selected for the analysis and their correspondence among all the gels had not been manually validated.

**Fig 3:**
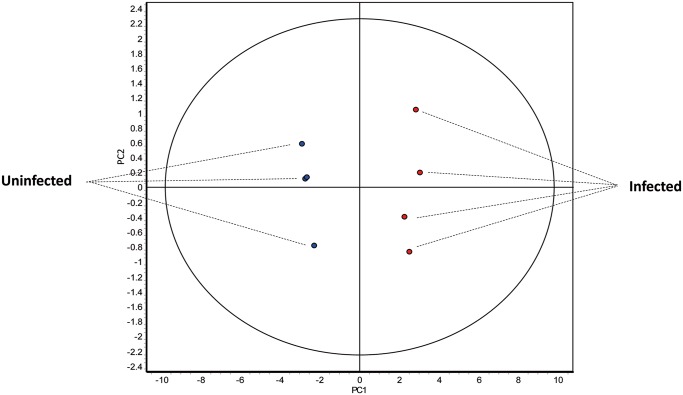
Multivariate statistical analysis applied to the set of 361 spots displaying greater significant statistical differences (*p*<0.01; *q*<0.05) in the 2D-DIGE experiment between the ileum of *Echinostoma caproni*-infected and uninfected mice. 2-dimensional score plot from the principal component analysis of the 8 individual biological replicates (*E*. *caproni*-infected in red and uninfected in blue).

**Fig 4:**
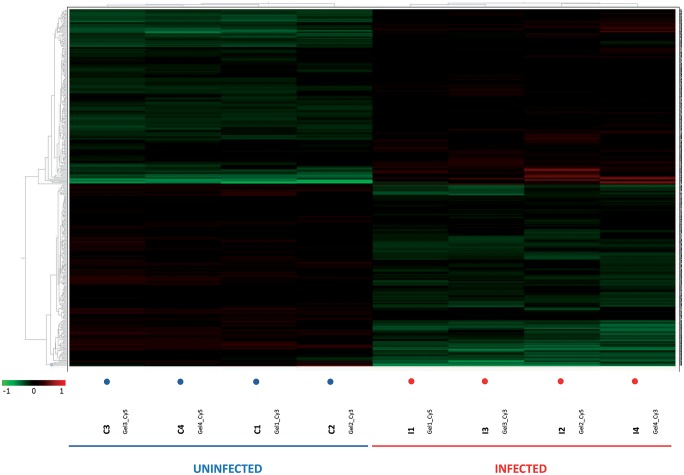
Heat-map with dendrograms from the hierarchical clustering analysis (Euclidean) obtained using the DeCyder extended data analysis module. Rows represent individual proteins and columns represent the individual biological replicates indicated at the bottom of the graph. The color in each cell represents the expression using a standardized log abundance scale ranging from negative values (green) to positive values (red).

As expected, when multivariate statistical tests were performed on the set of 361 spots differentially expressed between uninfected and infected mice (*p*<0.01 and *q*<0.05), both PCA and HCA also separated the biological replicates into two different groups. In the PCA the samples were separated by the first principal component, indicating that this set of proteins is enough to explicate the differences between the two groups (Fig 3). Similarly, HCA grouped replicates in two categories each including infected or uninfected samples (Fig 4). In this case, the protein spots were also classified in two main categories according to their expression pattern, i.e. up- or down-regulated in one group of samples respect the other one (Fig 4). This confirms that, *E*. *caproni* significantly alters the protein expression pattern of the IEC, affecting a large number of proteins 2 weeks after the infection.

### Identification of the most differentially expressed proteins in the ileum of infected mice and the cellular processes that were consequently affected

In view of the large number of proteins significantly affected by the *E*. *caproni* infection, those spots displaying a greater difference between uninfected and infected animals were selected for identification by MS and database search. A total of 37 from 47 spots with *p*<0.01, *q*<0.05 and AVR≥|2| were accurately identified: 11 of them overexpressed in the ileum of infected mice and 26 down-regulated as a consequence of the infection (S1 Fig). These 37 spots corresponded to 31 different proteins (10 up-regulated and 21 down-regulated), since 6 redundancies were detected (Table 1). This can be attributed to different post-translational modifications, the existence of isoforms or to protein modifications during sample preparation [24]. Identified proteins are classified in Table 1 according to their function, with detailed information comprising accession number, 2D-DIGE-related data, cellular role, localization and identification parameters.

**Table 1:** Proteins identified by 2D-DIGE as the most differentially expressed in the ileum of mice two weeks post *Echinostoma caproni* infection.

Spot^a^	Protein	Species (GI)^b^	AVR^c^	*t* test^d^	Cellular role	Loc^e^	Identified by	Cov. (%)^f^	MASCOT *score*
	**Metabolism**								
1032	Pyruvate dehydrogenase X component	*Mus musculus* (28201978)	-2.03	0.0053	Glycolisis-TCA cycle link	Mit	LC-MS/MS	24.7	278
1043	4-trimethylaminobutyraldehyde dehydrogenase	*M*. *musculus* (78099319)	-2.34	0.0023	Amine and polyamine biosynthesis Carnitine biosynthesis	Cyt	LC-MS/MS	54.5	596
1064	4-aminobutyrate aminotransferase	*M*. *musculus* (37202121)	-3.39	0.0013	GABA metabolism	Mit	LC-MS/MS	51.0	543
1172	Enolase 1B	*M*. *musculus* (70794816)	2.74	0.0023	Glycolysis Plasminogen activation	Cyt PM	LC-MS/MS	33.2	162
1183	Enolase 1B	*M*. *musculus* (70794816)	2.00	0.0047	Glycolysis. Plasminogen activation	Cyt PM	LC-MS/MS	66.4	567
1232	Elongation factor Tu	*M*. *musculus* (27370092)	-2.87	0.0012	Protein biosynthesis	Mit	LC-MS/MS	55.5	318
1237	Elongation factor Tu	*Mesocricetus auratus* (298351659)	-3.56	0.0012	Protein biosynthesis	Mit	MS/MS	71.0	88
1247	Ornithine aminotransferase	*M*. *musculus* (8393866)	-2.64	0.0091	Ornithine metabolism	Mit	MS/MS	33.0	106
1254	Ornithine aminotransferase	*M*. *musculus* (8393866)	-2.14	0.0076	Ornithine metabolism	Mit	LC-MS/MS	45.6	669
1259	Ornithine aminotransferase	*M*. *musculus* (8393866)	-3.94	0.0023	Ornithine metabolism	Mit	LC-MS/MS	44.2	738
1342	Aminoacylase-1	*M*. *musculus* (13384746)	2.23	0.0027	Amino acid metabolism	Cyt	LC-MS/MS	45.3	267
1556	Isocitrate dehydrogenase 3 (NAD+) alpha	*M*. *musculus* (148693875)	-2.15	0.0074	TCA cycle	Mit	MS/MS	33.0	117
1617	Ornithine carbamoyltransferase	*M*. *musculus* (129277)	-2.26	0.0079	Amino acid biosynthesis	Mit	MS/MS	29.0	81
1712	L-lactate dehydrogenase A chain	*M*. *musculus* (6754524)	2.70	0.0059	Pyruvate fermentation to lactate	Cyt	LC-MS/MS	50.0	383
1791	Hydroxymethylglutaryl-CoA lyase	*M*. *musculus* (171543858)	-3.42	0.0028	Ketogenesis	Mit	LC-MS/MS	41.5	327
1920	Sulfotransferase family 1B, member 1	*M*. *musculus* (148706035)	2.34	0.0029	Lipid and steroid metabolism	Cyt	LC-MS/MS	28.2	168
2206	Enoyl-CoA hydratase, short chain	*M*. *musculus* (148685962)	-2.00	0.0053	Fatty acids β-oxidation	Mit	LC-MS/MS	40.3	320
3412	Cytochrome c oxidase subunit IV isoform 1	*M*. *musculus* (148679695)	-2.52	0.0013	Mitochondrial electron transport chain	Mit	LC-MS/MS	42.2	100
	**Electron transport**								
1865	Electron transferring flavoprotein, alpha polypeptide	*M*. *musculus* (13097375)	-2.91	0.0012	Electron carrier	Cyt	LC-MS/MS	53.8	335
1869	Electron transferring flavoprotein, alpha polypeptide	*M*. *musculus* (13097375)	-2.21	0.0042	Electron carrier	Cyt	LC-MS/MS	58.9	547
2185	Electron transfer flavoprotein subunit beta	*M*. *musculus* (38142460)	-2.16	0.0016	Electron carrier	Cyt	LC-MS/MS	71.0	477
2186	Electron transfer flavoprotein subunit beta	*M*. *musculus* (38142460)	-2.20	0.0027	Electron carrier	Cyt	LC-MS/MS	39.0	166
	**Structural proteins**								
2192	Lamin B	*M*. *musculus* (293689)	-2.16	0.0029	Nuclear lamina component: involved in nuclear membrane architecture, chromatin organization, signaling…	Nuc	LC-MS/MS	60.4	374
2219	Keratin, type II cytoskeletal 8	*M*. *musculus* (114145561)	-2.87	0.0031	Intermediate filaments: structural activity	Cyt	LC-MS/MS	23.1	222
2940*	Keratin, type I cytoskeletal 19	*M*. *musculus* (6680606)	2.89	0.0035	Intermediate filaments: structural activity	Cyt	LC-MS/MS	28.0	14.43
	**Metal-binding proteins**								
1490	Zinc-binding alcohol dehydrogenase domain-containing protein 2	*M*. *musculus* (31559926)	-2.69	0.0082	Oxidoreductase activity	Px	LC-MS/MS	18.3	143
2124	Haloacid dehalogenase-like hydrolase domain-containing protein 3	*M*. *musculus* (21312204)	-2.38	0.0097	Hydrolase activity	Mit	MS/MS	26.0	89
2247	Fumarylacetoacetate hydrolase domain containing 1	*M*. *musculus* (20072495)	-2.32	0.0059	Hydrolase activity	Mit	LC-MS/MS	44.9	139
	**Lipid-binding proteins**								
2339	Apolipoprotein A-I, isoform CRA_b	*M*. *musculus* (148693731)	3.45	0.0033	Lipid transport	Secreted (Golgi)	LC-MS/MS	42.4	197
3615	Fatty acid-binding protein, intestinal	*M*. *musculus* (6679737)	3.20	0.0012	Intracellular lipid trasnport	Cyt	LC-MS/MS	43.9	124
3663	Fatty acid-binding protein, liver	*M*. *musculus* (8393343)	2.54	0.0024	Intracellular lipid transport	Cyt	LC-MS/MS	59.8	91
	**Protein-binding proteins**								
895	Protein disulfide-isomerase A3 precursor	*M*. *musculus* (112293264)	-2.27	0.0059	Cell redox homeostasis Protein folding Signaling	ER Cell surf.	LC-MS/MS	56.5	762
2007	Proteasome subunit alpha type-1	*M*. *musculus* (33563282)	-2.12	0.0049	Proteolysis	Cyt	LC-MS/MS	48.7	336
	**Detoxifying/Antioxidant proteins**								
1020	Aldehyde dehydrogenase	*M*. *musculus* (21312260)	-2.74	0.0017	Alcohol metabolism	Mit	LC-MS/MS	22.0	122
2486	Manganese superoxide dismutase	*M*. *musculus* (53450)	-2.12	0.0015	Destruction of superoxide anion radicals	Mit	LC-MS/MS	30.6	181
	**Calcium-binding proteins**								
3277	EF-hand domain-containing protein D2	*M*. *musculus* (31981086)	2.36	0.0012	Cytoskeleton associated adaptor protein	Mb raft	LC-MS/MS	30.2	113
	**Sugar-binding proteins**								
3621	Galectin-2	*M*. *musculus* (269914146)	2.20	0.0033	Lectin	Cyt/ECM	MS/MS	32.0	142

TCA, tricarboxylic acid cycle; GABA, gamma-aminobutyric acid; Mit, mitochondrion; Cyt, cytoplasm; PM, plasma membrane; Nuc, nucleus; Px, peroxisome; ER, endoplasmic reticulum; Cell surf., cell surface; Mb raft, membrane raft; ECM, extracellular matrix; LC-MS/MS, liquid chromatography and tándem mass spectrometry; MS/MS, mass spectrometry.

^a^ The spot number corresponds to the number assessed to each spot in the 2D reference gel for DIGE data analysis and refers to the numbers in S2 Fig.

^b^ GI accession number in NCBI Protein database

^c^ Average volume ratio quantified by DeCyder BVA module

^d^ *p* value of Student’s *t* test calculated by DeCyder BVA module

^e^ Subcellular localization according to UniProt database

^f^ Percentage of amino acid sequence coverage for the identified proteins

* The protein corresponding to spot number 2940 could not be properly identified by MASCOT 2.5, but it was successfully identified using ProteinPilot v4.5 search engine (Paragon algorithm) with the following parameters: trypsin specificity, iodoacetamide cysteine-alkilation, taxonomy restricted to Metazoa and the search effort set to rapid. Both coverage (%) and score values indicated for this protein are ProteinPilot values (a score > 2.0 means that it was identified with confidence ≥ 99%).

**Fig 5:**
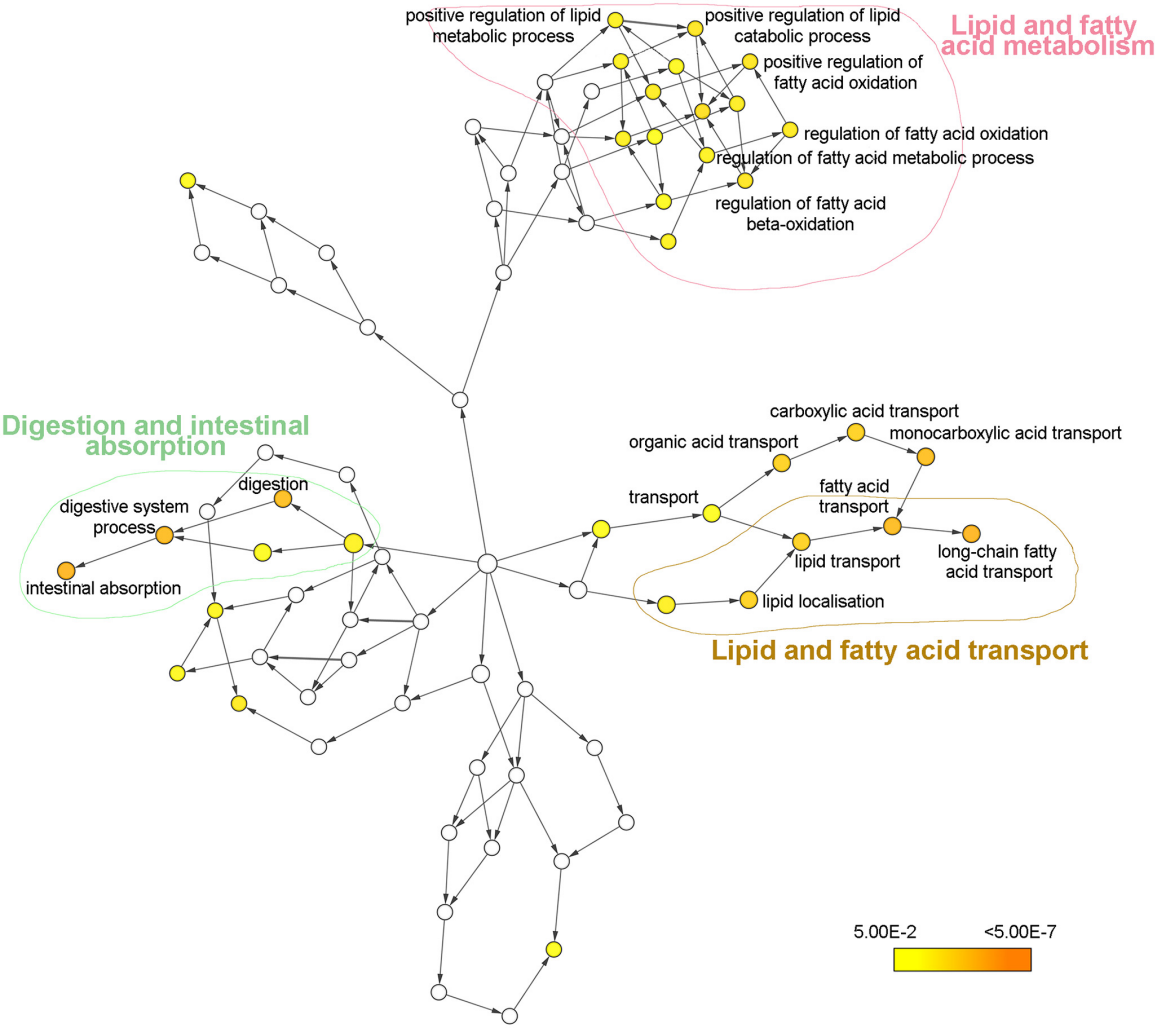
Biological process network for proteins with significantly up-regulated expression levels in the intestine of *Echinostoma caproni* infected mice. Node size is related to the number of proteins associated with a GO term, while color relates to the P-value for the statistical significance of the enrichment of a GO term.

**Fig 6:**
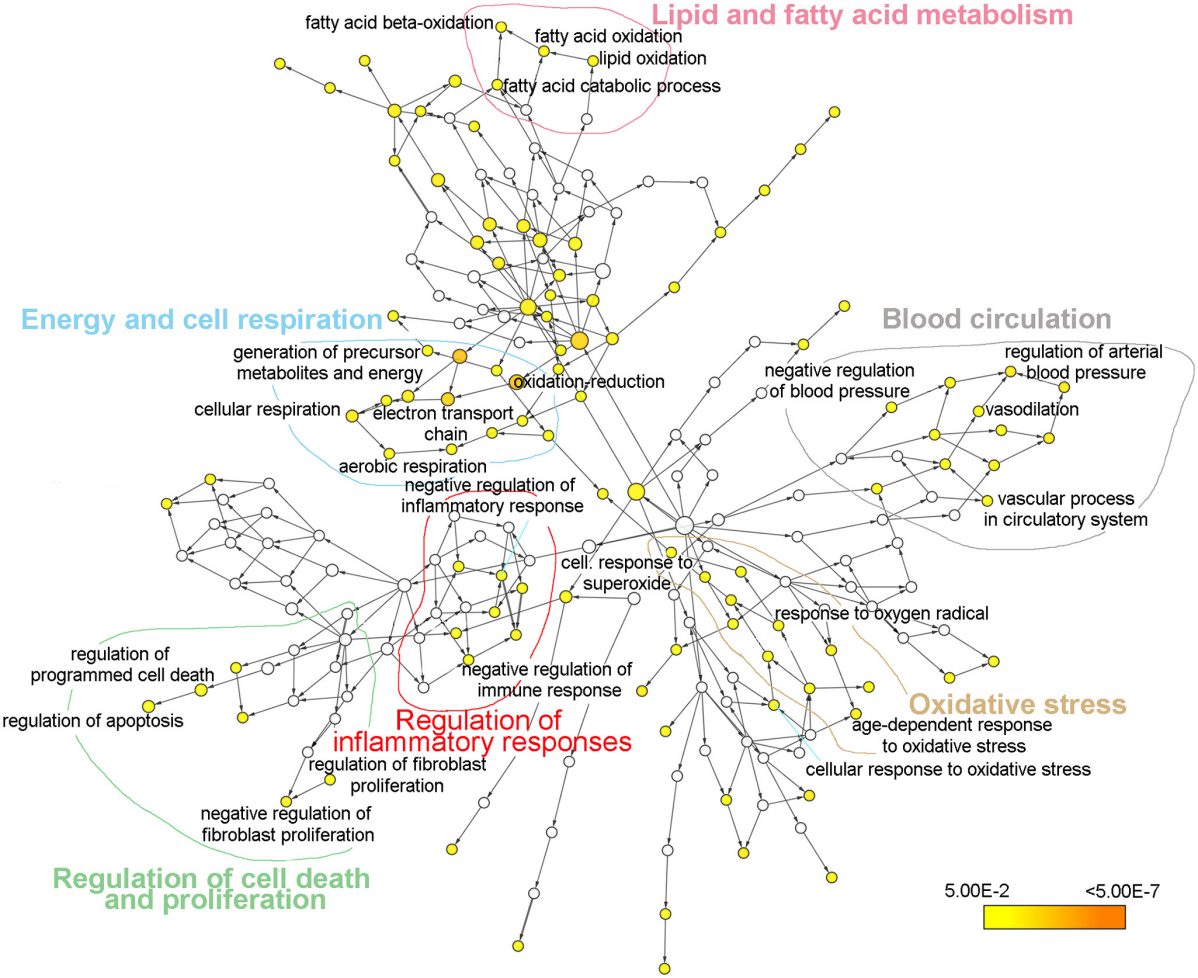
Biological process network for proteins with significantly down-regulated expression levels in the intestine of *Echinostoma caproni* infected mice. Node size is related to the number of proteins associated with a GO term, while color relates to the P-value for the statistical significance of the enrichment of a GO term.

An analysis of the GO biological process of the proteins presenting an up-regulated or down-regulated expression was performed using the plugin BiNGO with Cytoscape. Proteins overexpressed in the ileum of infected mice were related to three main processes: Lipid and fatty acid metabolism, lipid and fatty acid transport and digestion and intestinal absorption (Fig 5). In contrast, proteins with a down-regulated expression in the intestine of infected mice were related to different biological processes such as energy and cell respiration, regulation of inflammatory responses, oxidative stress and lipid and fatty acid metabolism among others (Fig 6).

## Discussion

### *E*. *caproni* chronic infection induces mitochondrial dysfunction and altered IEC metabolism

After a manual annotation of the proteins using the Uniprot database, the group of proteins that became more altered as a consequence of the infection was related to the energy metabolism. Our proteomic data suggests that mitochondrial function (particularly energy and cell respiration processes) is markedly reduced in the ileum of *E*. *caproni*-infected mice (Fig 6). Significant down-regulation of a component of pyruvate dehydrogenase complex (PDH) and the subunit α of NAD+-dependent isocitrate dehydrogenase 3 (IDH3) was detected (AVRs: -2.03 and -2.15, respectively). The mitochondrial PDH complex catalyzes the conversion of pyruvate to acetyl coenzyme A (acetyl-CoA), linking glycolysis to Krebs cycle. IDH3 is a mitochondrial matrix enzyme that catalyzes the rate-limiting step of the Krebs cycle, the oxidation of isocitrate to oxalosuccinate. Alteration of these processes deteriorate mitochondrial ATP production causing energy depletion [25]. In the case of an inefficient oxidative phosphorylation, the mitochondrial fat oxidation pathway becomes important in providing an alternative source of energy. The β-oxidation of fatty acids appears to be also affected in the IEC isolated from infected mice since enoyl-CoA hydratase, a mitochondrial enzyme that catalyzes the second step of each cycle of β-oxidation, was down-regulated after infection (AVR: -2.00). Moreover, fatty acids metabolism and, consequently, energy production were affected as a result of a reduction in the carnitine biosynthetic pathway. Carnitine is required for energy metabolism since it enables activated fatty acids to enter the mitochondrial matrix. In the ileum of *E*. *caproni*-infected mice, reduced expression of 4‐trimethylaminobutyraldehyde dehydrogenase, an enzyme involved in carnitine biosynthesis that catalyzes the conversion of 4-trimethylaminobutirate to γ-butirobetaine, was also noted (AVR: -3.39). Although the last step of carnitine biosynthesis from γ-butirobetaine occurs in the liver, precursor metabolites are absorbed in intestine and kidneys and transformed into γ-butirobetaine that is converted into carnitine [26]. Thus, reduced intestinal biosynthesis of carnitine-precursor metabolites affects mitochondrial import of fatty acids, reducing β-oxidation and favoring their cytosolic accumulation. This is supported by the fact that fatty acid binding proteins (FABPs) and apolipoprotein (Apo) A-I are overexppressed in the ileum of infected mice.

In the small intestine, both liver and intestinal FABPs (LFABP and IFABP, respectively) are expressed in villus enterocytes. In the ileum of *E*. *caproni*-infected mice both IFABP (AVR: +3.20) and LFABP (AVR: +2.54) overexpression occurs concomitantly with mitochondrial dysfunction and down-regulation of enoyl-CoA hydratase. In IEC, FABPs have been also proposed to have a role in the regulation of intracellular levels of unbound fatty acids [27–29], which can be toxic for the cells [30]. In our study, their overexpression may be a collateral consequence of the reduced mitochondrial metabolism and the subsequent accumulation of fatty acids in the cytosol of enterocytes. Increased expression of Apo A-I, the major protein component of high-density lipoprotein (HDL), was also detected in the ileum of infected mice (AVR: +3.45). The intestine can also act as a source of Apo A-I [31], which is incorporated to form mature chylomicrons that transport the exceeding lipids to other tissues such as adipose, cardiac or skeletal muscle [32].

All these metabolic alterations suggest that in the ileum of infected mice, enterocytes display a limited respiration and ATP production through the oxidative phosphorylation system, which can induce a metabolic shift to obtain energy from alternative metabolic processes. Indeed, parallel to mitochondrial dysfunction, lactate dehydrogenase (LDH) overexpression was detected in the ileum of infected mice (AVR: +2.70). LDH up-regulation associated to PDH down-regulation has been previously described [33,34] and involves a crucial shift in cell metabolism to prevent pyruvate accumulation and the consequent stop of glycolysis. This shift in the cellular energy supply is a signature of oxidative stress-induced cellular senescence [35]. Since oxidative phosphorylation is a more efficient mechanism for ATP production than anaerobic metabolism, such change in energy metabolism is normally accompanied by an increase in the glycolytic flux [33–35]. Our results revealed a simultaneous increase in the expression of two different isoforms of the glycolytic enzyme enolase 1, also named α-enolase, (AVRs: +2.00 and +2.74, respectively).

Mitochondrial dysfunction is associated with a number of medical disorders and ageing and may be a major mechanism underlying the development of mitochondria-related diseases consisting in an increase in the intracellular oxidative stress [36]. Increased production of reactive oxygen and nitrogen species (ROS and RNS, respectively) lead to an elevation in nitroxidative stress, which can oxidatively damage mitochondrial DNA, lipids and, primarily, proteins and, ultimately, induce tissue injury and cell death [36]. The establishment of chronic *E*. *caproni* infections in mice is known to be associated with the development of early and strong local inflammatory responses and tissue damage concomitantly with elevated mRNA levels of IFN-γ and iNOS [15,16].

Increased ROS production is commonly associated with failings in the mitochondrial respiratory chain that reduce effective oxidative phosphorilation and increase the leakage of electrons and the formation of reactive species [36]. According to our proteomic data two different isoforms of both α and β subunits of the electron transfer flavoprotein (ETF) were found to be down-regulated after infection (AVRs from -2.16 to -2.91), which is likely to be related to the infection-induced impairment of mitochondrial metabolism.

Furthermore, decreased expression of the cytochrome c oxidase (CcO) subunit IV isoform 1 (IV-1) was observed in the ileum of infected mice. Although CcO activity can be regulated at several levels, the subunit IV has been shown to be a key regulatory subunit in response to ATP and O_2_ levels [37]. At high ATP demand CcO IV-1 can be replaced by isoform 2 (IV-2) at the expense of ROS production [38]. Thus, the down-regulation of CcO IV-1 suggests that CcO activity is regulated through the modification of the expression of subunit IV isoforms in response to the infection. The gene expression of IV-2 is induced under hypoxic and toxic condition, and is up-regulated via hypoxia inducible factor 1 alpha (HIF-1α) [38,39]. In ischemic and inflammatory diseases of the intestine, the activation of HIF-1α in epithelial cells plays a protective role through the regulation of genes involved in the maintenance of epithelial tight barrier and mucosal immune response [40–42]. HIF-1α have been shown to correlate with PDH dysfunction and have a major role in promoting the shift of cell metabolism to anaerobic glycolysis [43], which agrees with our proteomic data. Although the mechanisms leading to inflammation-mediated hypoxia are not fully understood, most likely it involves vasculitis and edema [44]. Additionally, neutrophil migration into the intestinal mucosa is critical in depleting local O_2_ and activating HIF-1α [42]. Neutrophil infiltration at the site of the infection is also characteristic in the high-compatible host [15,16]. Therefore, in the ileum of infected mice, inflammation and neutrophilia may lead to overexpression of HIF-1α that, in turn, contributes to shift the enterocyte metabolism and the exhibition of a senescent phenotype. All this suggests that HIF-1α can be a key mediator in regulating the metabolic changes and controlling the intestinal pathology in response to *E*. *caproni* infection in mice, which we consider to merit further attention in future studies.

Finally, ROS accumulation would be favored by the down-regulation of the antioxidant enzyme manganese superoxide dismutase (MnSOD), a mitochondrial matrix and intermembrane space protein that transforms the highly reactive O_2_^·−^ into H_2_O_2_ and O_2_. Inactivation of MnSOD gene in mouse induces mitochondrial disease associated with ROS toxicity and apoptosis [45]. Although antioxidant enzymes are generally believed to be up-regulated in response to an oxidative stress [46], a positive role for reduced expression of MnSOD in uninfected ling the homeostatic dysregulation of the intestinal tissue during *E*. *caproni* chronic infection is discussed below.

Overall, the infection-induced alterations on the IEC metabolism suggest that *E*. *caproni* infection induces a rapid and intense mitochondrial dysfunction, mainly characterized by the shift of cell metabolism to an anaerobic use of glucose. These changes seem to be consequence of the oxidative stress induced by the overexpression of IFN-γ and iNOS in the intestinal mucosa of infected mice.

### *E*. *caproni* infection promotes early IEC hyper-proliferation and epithelial restitution

Intestinal tissue hyper-proliferation is a hallmark response to *E*. *caproni* chronic infection [18], and the results obtained herein strongly support this observation. *E*. *caproni* infection in mice is characterized by an intense tissue damage in the ileum caused by both the parasites and the local inflammatory response developed against the infection [15–17]. Moreover, villi tip erosion and gaps in the epithelial line are common at the site of infection [15,17]. Despite these facts, tissue necrosis is not developed suggesting that epithelial restitution mechanisms work actively in an attempt to restore the constant tissue damage. Herein, we have seen that *E*. *caproni* infections in mice induce alterations in several proteins implicated in the IEC proliferation and epithelial restitution (Fig 6).

Galectin 2 (Gal2) was found to be overexpressed in the ileum of infected mice (AVR: +2.20). Galectins are a family of lectins play a major role in re-epithelialization of wounded tissues [47–51]. In addition to Gal2, an EF-hand domain containing protein (EFhd2, also named swiprosin-1) was among the most up-regulated proteins in the ileum of infected mice (AVR: +2.36). EFhd2 is also up-regulated under inflammatory conditions [52]. EFhd2 is found together with actin and actin-binding proteins modulating bundling and cell spreading [53] and actin remodeling [54], respectively. During epithelial restitution, an extensive reorganization of the actin cytoskeleton is needed [55], suggesting that EFhd2 may play a role in this process. Both IFN-γ and nitric oxide (NO) have been shown to impair IEC migration through different mechanisms [56–58], thus Gal2 and EFhd2 appear as potential candidates to direct epithelial restitution under inflammatory conditions in the ileum of *E*. *caproni*-infected mice.

Once epithelial restitution has started, augmented cell proliferation is required to provide new enterocytes to restore the damaged area and the results obtained herein reveal that several pathways are involved in the regulation of tissue hyper-proliferation. The downregulation of the antioxidant enzyme MnSOD plays a role in this process (Fig 6). Apart from its function in controlling oxidative damage, MnSOD also plays a role on tissue renewal, since MnSOD genetic deficiency promotes cell turnover [59]. In our study, this downregulation may be one of the mechanisms responsible for infection-induced cell hyper-proliferation during the establishment of chronic infections. A striking feature is that changes in MnSOD expression are commonly accompanied by increased levels of ornithine decarboxylase (ODC). ODC was not found to be among the most altered proteins. However, three isoforms of the ornithine aminotransferase (OAT), which is involved in the catabolism of L-ornithine, the substrate of ODC, were found to be markedly down-regulated (AVR: -3.94, -2.64 and -2.14). Another enzyme involved in ornithine catabolism, ornithine carmaboyltransferase (OCT), was also down-regulated (AVR: -2.26). Ornithine is synthesized from arginine (Arg) by the cytosolic isoform of the enzyme arginase and is a necessary metabolite for the synthesis of polyamines and prolines. In addition to ornithine synthesis, Arg can be also metabolized by nitric oxide synthase (NOS) to generate NO and L-citrulline, so that arginase/NOS balance is determinant to displace Arg metabolism to one or another pathway [60]. As mentioned above, *E*. *caproni* infection in CD1 mice is characterized by increased mRNA expression of iNOS [16]. Hence, in the ileal epithelium of infected mice Arg metabolism can be expected to be displaced to the production of NO and L-citrulline at the expense of ornithine synthesis. Neither arginase nor iNOS appeared to be primarily affected during *E*. *caproni* infection in mice at proteomic level. The down-regulation of ornithine catabolic enzymes (i.e. OAT and OCT) may allow the increase in ornithine bioavailability for polyamine synthesis through ODC in milieu in which ornithine biosynthesis is diminished due to the displacement of Arg metabolism. Polyamines (putrescine, spermidine and spermine) are small, polycationic, organic molecules, synthesized from ornithine via ODC, which are mandatory to cell proliferation [61]. In inflammatory models, NO production is considered to be an early phase response, whereas the production of polyamines occur in the repair-phase response after iNOS inhibition by agmatine aldehyde [62]. However, in *E*. *caproni* chronic infections, both iNOS overexpression and crypt-cell hyper-proliferation occur early and exacerbate over the course of the infection [16,18]. Thus, the increase of ornithine bioavailability for polyamine synthesis through the down-regulation of enzymes responsible for its use in other metabolic pathways may represent a different route to guarantee tissue repair in the presence of sustained elevated levels of NO production during chronic infections.

We also have found increased expression of several proliferation markers, such as keratin (K19) (AVR: +2.89), aminoacylase 1 (ACY1) (AVR: +2.23) and sulfotransferase (SULT)1B1 (AVR: +2.34) in addition to two isoforms of α-enolase (AVR: +2.74 and +2.00). K19 is a marker of the gut morphogenesis, as it is mainly expressed in proliferative crypts [63]. Its overexpression in the ileum of infected mice is consistent with the crypt hyperplasia developed [18]. Similarly, the activity of the cytosolic enzyme ACY1, responsible for the deacylation of α-acylated amino acid residues during intracellular protein catabolism, is greater in the crypt areas than in the villous portion of small intestine [64]. Although the biological function of SULT1B is not well defined, high levels of its mRNA expression are detected in human fetal small intestine [65]. Its overexpression suggests that it play a role in cell proliferation, differentiation and/or tissue structural organization in the small intestine.

Apart from its role as a glycolytic enzyme, α-enolase serves as a plasminogen receptor on the surface of a variety of cells activing plasmin [66,67]. Plasmin-enolase interactions are involved in promoting cell migration in pathophysiological processes, such as the inflammatory response, cell invasion and cancer metastasis [68,69]. mRNA expression of α-enolase increases in growing cells, but remains almost at an undetectable level in the stationery/resting/quiescent phase [70] and, at protein level, it was found to be around 2-fold in proliferating versus differentiated human keratinocytes [71]. In the gastrointestinal tissue, α-enolase overexpression has been found in *Helicobacter pylori*-infected gastric mucosa, both at mRNA and protein levels [70,72], as well as in ulcerative colitis [73]. In *E*. *caproni*-infected mice, the elevated expression of α-enolase in the ileal enterocytes may well be a marker of intestinal inflammation and/or tissue overproliferation. This protein was also found to be overexpressed in the ileum of *E*. *caproni*-infected rats [21] in which increased epithelial cell renewal occurs in the absence of inflammatory responses [16–18], suggesting that α-enolase may have a key role in restoring homeostasis of injured intestine.

### Infection-induced enhanced cell death as a preventive mechanism for homeostatic dysregulation and malignancy

We have also found that several proteins implicated in the regulation of cell death became altered in the ileum of mice after *E*. *caproni* infection (Fig 6). In particular, proteins related to the mitochondrial-driven apoptotic pathway were affected, suggesting that the intrinsic pathway is activated because of the infection. This is of importance since in a context with increased cell proliferation, elevated levels of cell death are required to maintain tissue homeostasis and prevent massive dysregulation [74]. Moreover, prolonged inflammation and wound healing represent a high risk for DNA damage and malignant transformation and defective cells need to be rapidly eliminated [75].

Among the down-regulated proteins implicated in cell growth and apoptosis, we found MnSOD. It has been previously shown that MnSOD deficiency increases cell turnover via AP-1- p53-mediated pathways [59]. Moreover, the down-regulation of proteasome subunit alpha type 1 (PSMA1) may also affect the levels of p53. PSMA1 plays a role in gating the entry of proteins into the proteasome and its overexpression has been involved in tumor genesis [76,77]. PSMA1 is an important regulator of proteasome-mediated proteolysis, with a key role in cancer development and/or progression through modulation of p53 and nuclear factor kappa-light-chain-enhancer of activated B cells (NF-κB) signaling, which can play a key role in the control of intestinal tissue hyperplasia and homeostatic dysregulation after *E*. *caproni* infection. Proteasome inhibition has been associated with increased intrinsic apoptosis by different mechanisms. The availability of p53 increases since this protein is degraded through the ubiquitin-proteasome pathway [78,79]. Moreover, proteasome dysfunction also affects NF-κB through the stabilization of the inhibitory subunit IκB-a [80]. PSMA1 down-regulation has been detected in colorectal cancer cells after treatment with caffeic acid phenethyl ester [81] and this has been shown to induce apoptosis of cancerous cells through the inhibition of NF-κB signaling [82,83].

As mentioned before, the decreased expression of MnSOD in a milieu of elevated oxidative stress is surprinsing. However, it has been shown that this enzyme is transcriptionally regulated by NF-κB [84]. Suppression of NF-κB translocation results in reduction of MnSOD expression leading to ROS accumulation and cell death [85]. Therefore, the down-regulation of this antioxidant enzyme observed herein may be consequence of NF-κB repression and is likely to play a positive role in the ileum of infected mice, promoting ROS-mediated programmed cell death to counteract homeostatic dysregulation.

Alterations in different structural proteins were also noted. Together with the augmented expression of crypt-specific K19, a decrease in the expression of type II cytokeratin 8 (K8) was detected (AVR: -2.87). Keratins are structural proteins that associate to form non-covalent tissue specific heteropolymers (i.e. type I and type II keratins) that build up the intermediate filament cytoskeleton of epithelial cells. K8 is the main type II keratin present in mature enterocytes from the opening of the crypts to the villi apices [86]. K8^-/-^ mice showed a lack of intermediate filament cytoskeleton in small IEC, with differentiated enterocytes displaying progressive loss of apical membrane-associated proteins and alterations in microtubule organization [87]. Although tissue functional deficiencies were not observed in K8^-/-^ mice, it was noted that mature IEC lacking intermediated filament cytoskeleton displayed shortened microvilli and they seem to be unable to fully recover from tissue injury [87]. Moreover, small intestine enterocytes from K8^-/-^ mice appear to be more predisposed to apoptosis compared to those obtained from K8^+/+^ mice [88]. In the ileum of *E*. *caproni*-infected mice, this cytoskeletal deficiency may convert the infected epithelium even more sensitive to both parasite- and immune-mediated tissue damage, accelerating the induction of cell death.

A marked downregulation of the structural nuclear protein lamin B was also observed in mice after *E*. *caproni* infection (AVR: -2.16). Lamins, A- and B-types, are the major components of the nuclear lamina and, in addition to their role as structural proteins, these type V intermediate filament proteins contribute to nuclear envelope integrity [89]. A number of studies have linked B-type lamins to several aspects of cell physiology such as transcription, replication, spindle assembly, chromatin organization, resistance to oxidative stress or regulation of cell senenscence [90–93]. The decrease in lamin B1 expression occurs in response to stimulation of either p53 or pRB tumor suppressor pathways and induces inhibition of proliferation and premature senescence [92,93]. However, altered lamin expression is common in gastrointestinal neoplasms and reduced expression of either lamin A/C or lamin B1 is a marker of potential malignancy in the gastrointestinal tract in humans [94], thus their role in the infection with *E*. *caproni* needs to be further characterised. Finally, the expression of two isoforms of the mitochondrial elongation factor Tu (TUFM) was also found to be down-regulated in IEC from infected mice (AVR: -2.87 and -3.56, respectively). TUFM is one of the major mitochondrial biogenesis regulating proteins, which has been found to be down-regulated during ageing in muscle cells [95]. Moreover, its inhibition induces mitochondrial dysfunction and increased cell death in cancer cells [96], which is fully consistent with the results observed herein.

Despite the fact that proteomic data strongly support the idea of elevated levels of IEC death, tissue hyperplasia develops in the ileum of *E*. *caproni* infected mice [18], thereby suggesting that mitochondrial dysfunction and premature cellular senescence are not enough to equilibrate cell proliferation and death rates. In primary cultured hepatocytes, it has been shown that high amounts of IFN-γ-induced ROS are not sufficient to induce cell death, but a combination of ROS and proper endoplasmic reticulum (ER) stress responses is required to induce apoptosis [97]. In this sense, we have found protein disulfide isomerase A3 (PDIA3, also known as ERp57 or 1,25D_3_-MARRS) to be down-regulated in the ileum of infected mice (AVR: -2.27). PDIA3 is a stress-responsive protein, which is involved in protein folding, glycoprotein quality control and the assembly of the major compatibility complex class I in the ER [98]. Therefore, the lack of proper ER stress responses may be responsible for the low rate of IEC death and the development of tissue hyperplasia, despite premature senescence is induced in mature enterocytes in response to the infection. Nevertheless, in addition to the ER, PDIA3 is present in many other subcellular locations, which makes it difficult to predict the effects of its down-regulation over the course of the intestinal infection [98].

Constant wound repair represents an elevated risk for DNA damage and genomic instability in proliferating cells, promoting the development of a tumorigenic environment, with chronic inflammation being the most important risk factor [99]. Moreover, a continuous state of chronic inflammation and wound healing have been regarded as the key events for cancer development in other chronic helminth infections [100,101]. Our proteomic data suggest that both pro-tumorigenic (i.e. inflammation-mediated oxidative stress, cell hyper-proliferation) and anti-tumorigenic mechanisms (i.e. cellular senescence, apoptosis) are activated early after infection in *E*. *caproni*-infected mice. Malignant tumors are often developed at sites of chronic injury, re-epithelialization and inflammation. Thus, according to our results, persistent damage of the intestinal epithelium in long-lasting infections could represent a risk factor for cancer development.

### Infection-induced intestinal changes are host-dependent and appear to be crucial for the course of the infection

The proteomic alterations described herein can be directly associated with the chronic establishment of the parasite in hosts of high compatibility. These changes are markedly different to those observed in the ileum of infected rats, in which the parasite is rejected a few weeks after infection. A previous study [21] showed that the effects of E. caproni infection on the IEC of rats are low in comparison with mice, mainly inducing the overexpression of proteins related with the cytoskeleton and the maintenance of the functional integrity of the epithelial barrier (e.g. actin, T-plastin, both 8 and 19 cytokeratins or annexin A4). Consequently, changes on the absorptive/secretory function of enterocytes and, especially, an increased regenerative capacity of the intestinal epithelium appear to be potentially IL-13-mediated effector mechanisms involved in the early rejection of worms in rats. In contrast to mice, a strict control of proliferation and programmed cell death seems to be essential to maintain the intestinal homeostasis in rats, hence protecting the host against the injurious effects of the infection. This is consistent with the overexpression of the intestinal proliferation marker K19 and chaperones such as a heat shock cognate 71 KDa and BiP, together with the down-regulation of peroxiredoxin 3, prohibitin and 14-3-3 zeta isoform [21].

Moreover, proteomic data indicate that cellular energy metabolism becomes differentially modified in the ileum of E. caproni-infected mice and rats. Whereas in mice the intestinal infection induces mitochondrial dysfunction and an increase in the anaerobic use of glucose to yield ATP, in rats the transition to a more aerobic and oxidative metabolism is suggested, leading to a reduced glycolytic flux and overall ATP production [21]. These alterations in energy metabolism could be of relevance for a better comprehension of the mechanisms involved in the control of infections on mucosal surfaces.

In summary, our results indicate that the presence of the parasite induces a rapid and profound remodeling in the protein expression pattern of IEC, associated with the development of inflammation and oxidative stress. The identification of those proteins whose expression was mainly altered indicates that the cellular processes that become primarily affected after *E*. *caproni* infection in CD1 mice are related to the restoration of the damaged intestinal epithelium and the control of homeostatic dysregulation. Wound healing and crypt-cell hyper-proliferation appear to be constitutively active processes from the early stages of the infection. Concomitantly, mitochondrial dysfunction and cytoskeletal changes indicate that cellular senescence is induced on mature enterocytes. These facts, together with the pro-apoptotic changes observed, suggest that programmed cell death is augmented in the ileal mucosa of infected mice. Although previous studies have shown that proliferation and cell death are not well balanced in the ileum of infected mice, and the IEC turnover is diminished after infection, augmented cell death may be essential to control the level of homeostatic dysregulation in the gut and eliminate potentially damaged cells, which may conduct to malignant transformation.

## Supporting Information

S1 Fig Reference image of the 2D-DIGE gel, indicating the protein spots identified by mass spectrometry. Green squares indicate down-regulated spots 2 weeks after *Echinostoma caproni* infection and red squares show up-regulated spots. Identification details are shown in Table 1.
(TIF)

S2 Fig Heat-map with dendrograms from the hierarchical clustering analysis (Euclidean) obtained using the DeCyder extended data analysis module. Rows represent individual proteins and columns represent the individual biological replicates indicated at the bottom of the graph. The color in each cell represents the expression using a standardized log abundance scale ranging from negative values (green) to positive values (red).
(TIF)
