# Supplementary figures and images for: Altered Protein Expression in the Ileum of Mice Associated with the Development of Chronic Infections with Echinostoma caproni (Trematoda)
Source: PLoS Negl Trop Dis. 2015 Sep 21;9(9):e0004082. doi: 10.1371/journal.pntd.0004082 (PMC4577103; doi:10.1371/journal.pntd.0004082)

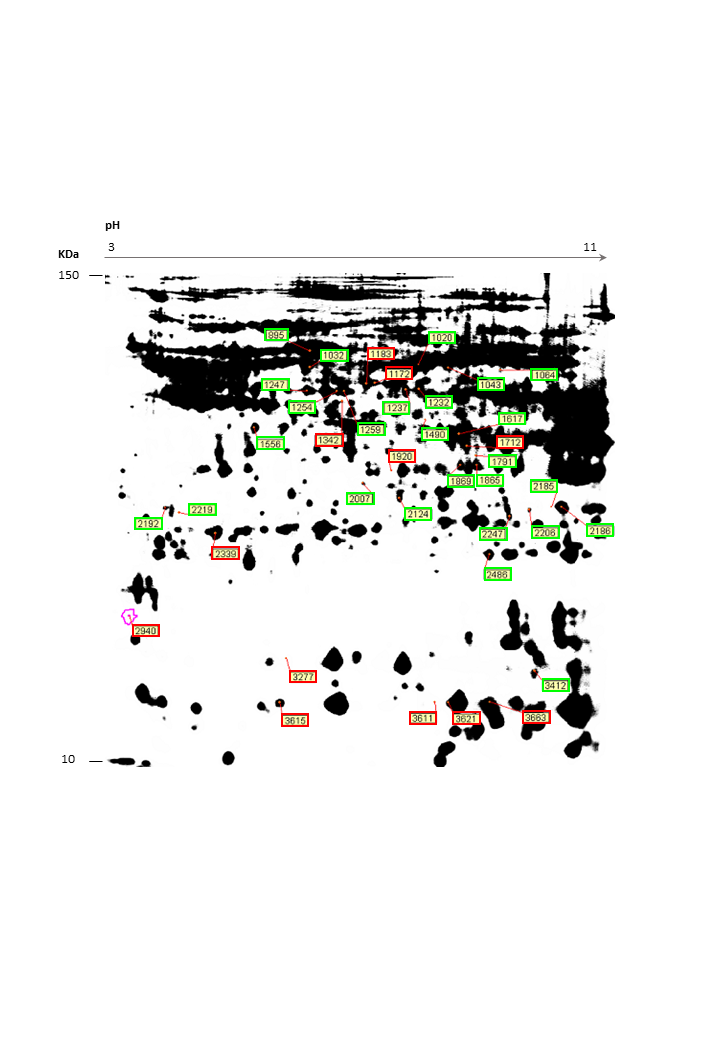

Supplement: S1 Fig — Green squares indicate down-regulated spots 2 weeks after Echinostoma caproni infection and red squares show up-regulated spots. Identification details are shown in Table 1. (TIF) [file pntd.0004082.s001.tif]

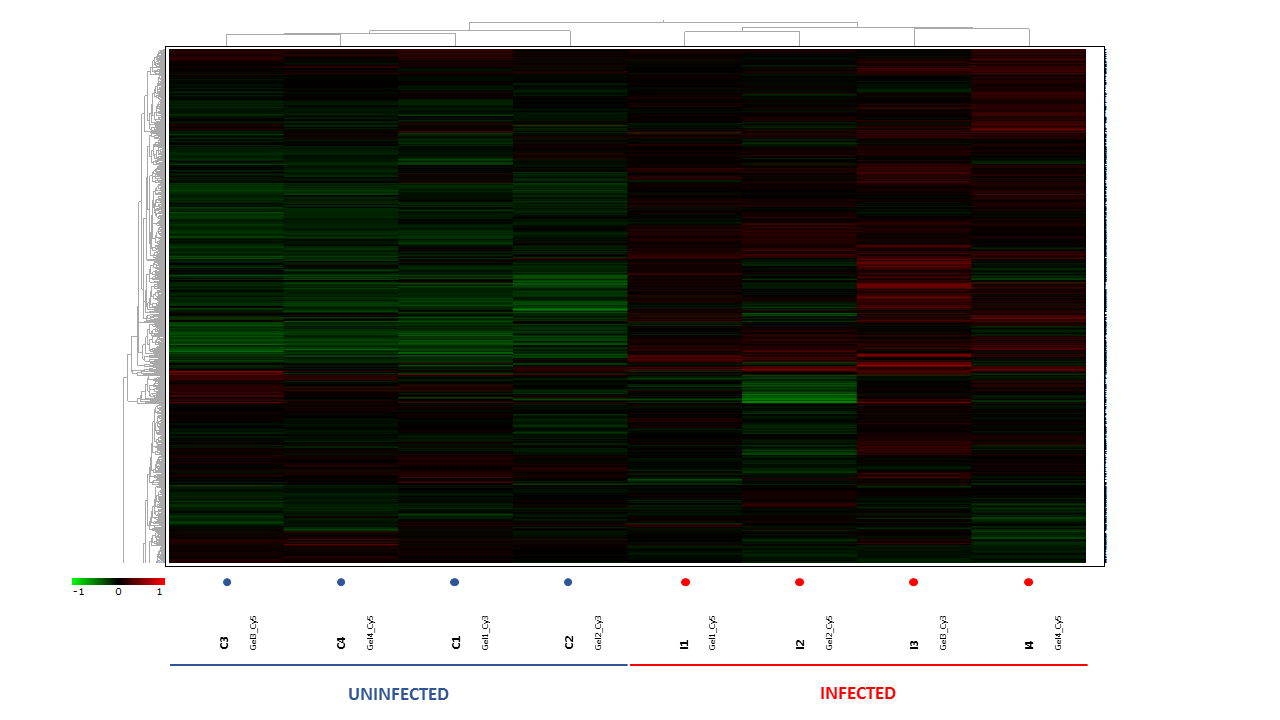

Supplement: S2 Fig — Rows represent individual proteins and columns represent the individual biological replicates indicated at the bottom of the graph. The color in each cell represents the expression using a standardized log abundance scale ranging from negative values (green) to positive values (red). (TIF) [file pntd.0004082.s002.tif]
